# Reply to Roesler et al. Comment on “Karaulic et al. Exploring Novel Applications: Repositioning Clinically Approved Therapies for Medulloblastoma Treatment. *Cancers* 2025, *17*, 3659”

**DOI:** 10.3390/cancers18050803

**Published:** 2026-03-02

**Authors:** Arthur Karaulic, Clémence Fournier, Gilles Pagès

**Affiliations:** University Côte d’Azur, Institute for Research on Cancer and Ageing of Nice (IRCAN, UMR CNRS 7284/U INSERM 1081), 06100 Nice, France; arthur.karaulic@univ-cotedazur.fr (A.K.); clemence.fournier@univ-cotedazur.fr (C.F.)

We gratefully thank R. Roesler, M. da Cunha Jaeger, C. Brunetto de Faria, and A. Thomaz [1] for their supportive comments on our article [2]. Our study focused on evaluating the potential for repositioning clinically approved treatments for pediatric medulloblastoma (MB). Patients with MB who relapse following conventional therapy—combining surgery, chemotherapy, and radiotherapy—have a particularly poor prognosis. Consequently, there is an urgent need to develop innovative therapeutic strategies.

However, the development of entirely new treatments is a lengthy and complex process. In contrast, repurposing existing drugs offers a more rapid and feasible alternative for these high-risk patients, as such agents have already undergone the full spectrum of regulatory toxicity testing required for de novo drug development. To this end, we employed an unbiased approach by screening molecular targets of drugs that have shown efficacy across various cancer types, regardless of whether they originated from solid or hematologic malignancies. Survival was used as our primary readout of therapeutic relevance.

Interestingly, while overexpression of several drug targets correlated with reduced overall survival (OS), others were unexpectedly associated with improved OS. These findings underscore the context-dependent and tumor-specific nature of gene function and therapeutic response. Among the most compelling targets identified was BCL2, making its clinically approved inhibitor venetoclax a promising candidate for therapeutic repositioning in MB. We subsequently demonstrated that venetoclax exhibits strong anti-tumor activity, both as a monotherapy and in combination with the standard-of-care chemotherapeutic agent etoposide. This choice was further supported by the extensive clinical experience with venetoclax, particularly in hematologic malignancies, making our findings both provocative and clinically relevant.

Our final screening table also highlighted several other promising targets. We were therefore pleased to receive the comments from Roesler and colleagues regarding the TRK receptors, an axis previously investigated in depth by their team [3,4]. We congratulate them on their elegant work, which independently validates our findings and reinforces the robustness and reproducibility of the results generated by both groups.

Together, these complementary datasets support the soundness of our approach and strengthen the rationale for developing innovative, targeted therapeutic strategies for medulloblastoma patients facing therapeutic dead ends.
